# The dental management of patients irradiated for head and neck cancer

**DOI:** 10.1038/s41415-023-5864-z

**Published:** 2023-06-09

**Authors:** Elizabeth Z. Goh, Nicholas Beech, Nigel R. Johnson, Martin Batstone

**Affiliations:** 821985008738976553450 Faculty of Medicine, University of Queensland, Brisbane, Queensland, Australia; 503489103095026720789 Faculty of Medicine, University of Queensland, Brisbane, Queensland, Australia; School of Dentistry, University of Queensland, Brisbane, Queensland, Australia; Oral and Maxillofacial Department, Princess Alexandra Hospital, Brisbane, Queensland, Australia; 754975814030875350667 Faculty of Medicine, University of Queensland, Brisbane, Queensland, Australia; Oral and Maxillofacial Department, Royal Brisbane and Women´s Hospital, Brisbane, Queensland, Australia

## Abstract

Patients undergoing radiotherapy for head and neck cancers are prone to a range of dental complications, including mucositis, trismus, xerostomia, radiation caries and osteoradionecrosis. Specific considerations include the preventive, restorative and rehabilitative management of such patients, and the prevention and treatment of complications. This article aims to highlight the current understanding and management of dental needs for patients who have had or will undergo radiotherapy.

## Key points

- Patients undergoing radiotherapy for head and neck cancers are prone to a range of dental complications, including mucositis, trismus, xerostomia, radiation caries and osteoradionecrosis.
- The multidisciplinary team plays a central role in the preventive, restorative and rehabilitative management of irradiated head and neck cancer patients.
- Further research is required to support decisions around pre-radiotherapy extractions, implant placement in irradiated bone, and the treatment and prevention of osteoradionecrosis.

## Introduction

Radiotherapy for head and neck cancers utilises ionising radiation to damage the genetic material of vulnerable malignant cells and cause cell death. Adverse effects arise from the same mechanisms damaging normal cells, especially those which are rapidly dividing or less capable of repair. In the oral cavity, these can be cells of the mucous membranes, underlying soft tissue, salivary glands, teeth, periosteum, bone and vasculature. This results in specific radiation syndromes: xerostomia from salivary gland injury; mucositis from epithelial damage; trismus from collagen structure changes; radiation caries from pathological alterations in tooth structure and normal flora; and osteoradionecrosis (ORN) from reduced bone healing capacity.

Management of oral health is paramount for head and neck cancer patients, as dental complications are common. Important considerations include specific preventive, restorative and rehabilitative management, and the prevention and treatment of complications. Since our first article,^1^ the past decade has seen increasing use of dental implants and landmark ORN clinical trials. In this article, we aim to provide an update on the current management of dental needs for patients who have had or will undergo radiotherapy.

## Pre-radiotherapy

### Dental assessment

A multidisciplinary team (MDT) approach to managing head and neck cancer patients is considered best practice.^2^ Given the oral implications of the disease and its treatment, the MDT should include dental practitioners with expertise in the preventive, restorative and rehabilitative management of these patients.^2^ MDT variations can be expected internationally.^2^ For example, in the UK, both pre-treatment assessment and post-surgical rehabilitation are performed by specialist restorative dentists in the MDT, while in Australia, this is commonly performed by experienced general dentists and specialist prosthodontists. While abiding by local standard care pathways is important, the principles of care remain the same.

Ideally, every patient receives a thorough pre-radiotherapy assessment with consideration of their diagnosis, prognosis, proposed treatment, individual factors and oral health status. This facilitates appropriate preventive care and any required immediate treatment, as well as comprehensive planning of the final rehabilitation from the beginning of treatment.

A full medical and dental history should be taken, as with all patients. Important aspects of the cancer diagnosis include tumour type and staging, location, and relationship to adjacent structures. Important treatment factors include anticipated radiation dose, field size and location, and any chemotherapy or surgery. Doses over 60 Grey, especially in areas involving the major salivary glands, increase the risk of complications. Motivation and ability to manage oral hygiene regimens are crucial but difficult to assess in the limited pre-radiotherapy phase. Current oral hygiene habits and prior engagement with oral health professionals may be helpful. Risk factor modification, such as smoking and alcohol cessation, should be discussed. Finally, a thorough clinical and radiographic examination should be performed, and a comprehensive treatment plan formulated. Unrestorable or periodontally hopeless teeth are extracted with minimal trauma before radiotherapy, irrespective of fields. All healthy teeth and deeply impacted teeth without pathology are retained. Patients are then maintained on three-monthly recalls, daily fluoride and bicarbonate rinses, and restorations as required. Primary dental care practitioners should be aware of their local MDT units and refer patients who present with complications.

### Prevention and restorations

The general goals of dental care in achieving a functional and aesthetic dentition are the same for head and neck cancer patients. Indeed, they are all the more relevant in considering the increased prevalence of dental disease in this population, with one study reporting that 80% of patients required pre-radiotherapy dental care, of which 60% required extractions.^3^ Importantly, the interval between the decision to treat and radiotherapy commencement is often short, so treatment must be prompt.

Scaling, prophylaxis and fluoride application should be performed. If required, simple restorations should be placed before radiotherapy begins, and provisional glass-ionomer cements (GICs) are often appropriate if definitive restorations are not possible in the given timeframe. Amalgams are generally avoided as they can cause back-scatter and subsequent local mucositis.^4^ Any sharp cusps or restorations should be smoothed or repaired to avoid trauma to the vulnerable irradiated soft tissues. Dentures should be checked to ensure they are well-fitting to avoid ulceration and patients should be advised to avoid use until radiotherapy is completed. Impressions should be taken for study models, and the fabrication of medicament trays or soft mouthguards at a later date.

### Extractions

Decisions regarding pre-radiotherapy extractions aim to avoid ORN while considering impacts on quality of life.^5^^,^^6^ Prospective evidence-based recommendations are lacking, likely because determining tooth prognosis is complex. Prognostication extends beyond the tooth to include highly variable factors, such as oral hygiene ability, nutrition and access to care, and can be modified by operator experience and patient risk tolerance. A helpful guide may be Ben-David *et al.*'s ten-year retrospective study, which found zero cases of ORN in all head and neck radiotherapy patients at their centre within six months.^7^ Their treatment protocol was: 'teeth with non-restorable caries, or caries that extend to the gum line, teeth with large, compromised restorations with significant periodontal attachment loss (pocketing >5 mm), and those with severe erosion or abrasion are extracted if they are in parts of the jaws expected to receive a high dose. Teeth residing in the anterior mandible are not considered for extraction unless the primary tumour was in the oral cavity. Decisions about extraction were significantly affected by the patient's competence and interest in performing meticulous oral hygiene, and by past history of dental service usage'.^7^

There is inconsistent evidence on the impact of pre-radiotherapy extractions on ORN risk. A 2021 meta-analysis reported an ORN incidence of 5.5% for pre-radiotherapy extractions and 5.3% for post-radiotherapy extractions, while acknowledging other factors, such as indication for extraction, surgical technique and radiation technique.^8^ Some studies indicate an increased ORN incidence with pre-radiotherapy extractions; while other studies report that lack of pre-radiotherapy extractions is a significant risk factor for ORN, possibly due to retaining dental or periodontal foci of infection which required post-radiotherapy extractions.^8^ For impacted third molars, the evidence is inconclusive for the pre-radiotherapy decision to extract or retain.^9^ Overall, clinical judgement in managing at-risk teeth is critical, and prophylactic extraction of healthy teeth based on location within the radiation field appear to be unjustified.

If extractions are performed, a two- to three-week waiting period for mucosal healing is acceptable before radiotherapy commencement, which should not be delayed for complete dentoalveolar bone remodelling. The latter will likely require several months and be occurring during radiotherapy. Hence, patients rendered edentulous through pre-radiotherapy extractions still have an increased risk of ORN compared to patients edentulous at initial presentation, who have an extremely low risk of ORN.^10^

## During radiotherapy

### Xerostomia, mucositis and trismus

Xerostomia results from damage to salivary glands, especially the parotids, hence affecting speech, taste, chewing and swallowing. Mucositis results from damage to the oral epithelium and can cause considerable pain and functional impairment. Both increase the risk of oral infections, such as caries and candidiasis, due to reduced defences from hyposalivation and epithelial breakdown. Thus, meticulous and gentle oral hygiene is key in management. For xerostomia, salivary substitutes, sialogogues (such as sugarless chewing gum or casein phosphopeptide-amorphous calcium phosphate [CPP-ACP] gum), and regular non-medicated oral rinses can provide symptom relief. For mucositis, adequate hydration, avoidance of irritants (such as tobacco and alcohol), and symptomatic strategies, including topical barrier gels and improving salivary flow, can be helpful. Appropriate analgesia and dietitian assessment of oral intake are also important considerations.

Trismus as a peri- or post-radiotherapy complication results from fibrosis of mastication muscles and restricts chewing, speech and access for oral hygiene. Early physical therapy, including jaw massage and exercises with input from the appropriate MDT specialist, is key to management. Maintaining good oral hygiene is also important.

### Emergencies

Comprehensive pre-treatment assessment and management of incipient dental conditions should minimise dental emergencies during radiotherapy. Radiotherapy interruptions should be avoided, as delays reduce treatment efficacy and thus survival.^11^ Early liaison with the radiation oncologist to discuss the treatment course is ideal. Acute toothaches may be managed with standard restorative or endodontic techniques, taking into consideration intraoperative challenges due to general discomfort, limited opening and mucositis. Extractions, where unavoidable, should have a low threshold for tertiary referral, especially for teeth in the radiation field.

## Post-radiotherapy

### Prevention and restorations

Radiation caries has a multifactorial aetiology. The primary cause relates to the diet, such as the use of sugar-containing oral nutritional supplements for disease-related malnutrition.^12^ Changes in salivary flow and composition predispose a more cariogenic microbiome, while trismus, mucositis and pain impair oral hygiene. One-in-three patients develop caries within two years of radiotherapy.^13^ Incidence is related to radiotherapy dose, with an odds increase of two to three at 30-60 Grey, and ten at over 60 Grey.^14^ It is thought that the salivary glands can withstand damage up to 30 Grey and sustain maximal damage at 30-60 Grey, with additional risk from direct radiation-induced biomechanical damage to tooth structure.^14^ Accordingly, the most common sites of radiation caries are the labial surfaces of cervical, cuspal and incisor areas, which tend to be the most caries-resistant areas in non-irradiated patients, despite being subject to compression, shear and torsional forces.^14^ Progression is typically aggressive and correlates poorly to clinical appearance.^15^

Prevention is key. Regular oral hygiene includes gentle and thorough brushing and flossing and non-acidic fluoride or bicarbonate mouthrinses. Daily topical fluoride in custom trays is recommended. Previous studies have found a 14% reduction in moderate-severe dental deterioration for each day of fluoride use a week^14^ and significantly improved root caries control with the daily use of CPP-ACP with fluoride.^16^

Challenges in the restorative management of irradiated patients include: trismus, which limits oral hygiene and intraoperative access; xerostomia, which reduces salivary protection; and radiation-induced structural alterations, which diminish the biomechanical properties of enamel and dentine and impair the bond strength of adhesive systems.^17^^,^^18^ An ideal restorative material would be caries-resistant, durable, adherent to tooth structure, aesthetic and easy to use. No current material meets this standard and only limited clinical data is available to guide material choice. A 2021 meta-analysis which defined restoration failure as recurrent caries and/or marginal/anatomic failure within two years post-radiotherapy found higher rates in composite resins (26-44%) than in GIC (0-7%) and resin-modified GIC (RMGIC) (11-26%). However, failure rates defined solely by marginal/anatomic failure were higher in GIC (74-100%) and RMGIC (67-78%) than in composite resins (33-41%).^19^ GIC and RMGIC may be more suitable in high-caries-risk situations, as they offer simpler bonding procedures, chemical adhesion, and fluoride release, which can minimise recurrent caries even with subsequent restoration loss. However, advances in material properties within the past two decades since these studies call for further research using contemporary materials.

Endodontic treatment for pulpally involved teeth is generally preferred to extraction. Even teeth with poor restorative prognosis can be root-filled and sealed to control symptoms and infection while minimising ORN risk. However, trismus can limit access for dental dam placement and instrumentation, and unconventional access cavities on labial/incisal surfaces may be an acceptable compromise.^20^ Additionally, xerostomia can cause intraoperative discomfort and conductivity issues during apex locator use, and the use of artificial saliva may be helpful. There is limited data on endodontic treatment in irradiated patients, but success rates seem to be acceptable.^21^^,^^22^

### Rehabilitation

Current literature on oral rehabilitation in irradiated patients with conventional fixed and removable prostheses is scarce, with recent research gravitating towards implant-supported prostheses. Simple indirect restorations with hygienic design, including supragingival margins, may be considered for patients with excellent oral hygiene and stable dentition. Crowns and bridges are otherwise avoided in xerostomic patients due to the increased caries risk. Removable prostheses can limit plaque control and traumatise tissues, which is an important consideration in a xerostomic environment with reduced salivary protection. However, if dentures are required for aesthetics or function, then hygienic design, impeccable oral and denture hygiene, and regular recall are critical. Well-fitting dentures rarely cause ORN.^23^ The recovery period between radiotherapy completion and denture provision may depend on the surface area of denture-bearing tissues, but denture provision within six months or after one year are unlikely to differ in complication rates.^24^

Implants placed in irradiated bone are more than twice as likely to fail as those in non-irradiated bone.^25^^,^^26^ A 2016 meta-analysis reported survival rates of 84% in irradiated bone and 95% in non-irradiated bone.^26^ Timing of placement and nature of the bone (native versus grafted) did not seem to be a significant risk factor, although this was based on observational studies with lack of control for confounding factors.^26^ Careful patient selection to optimise outcomes and detailed pre-operative discussion to manage expectations are critical for implant placement in general, and especially when considering the uncertainties for implants in irradiated bone.

### ORN and minor oral surgery

ORN is the necrosis of irradiated bone without evidence of tumour recurrence persisting for at least three months.^27^ The exact pathophysiology remains unclear but proposed mechanisms include Marx's 'three H' theory^28^ and Delanian and Lefaix's fibroatrophic theory.^29^

ORN associated with extractions has a 5-15% incidence rate.^27^ This has been decreasing since the 1990s with the advent of modern radiotherapy techniques and improved dental prevention.^27^ However, extractions of compromised teeth may be required even with good preventive care. An atraumatic technique with primary socket closure is vital. Recommendations such as limiting the number of extractions in a single visit and avoiding certain local anaesthetics require further investigation.^30^ Endodontic treatment should generally be performed where viable.

ORN associated with implant placement is an emerging concern, with a 3% incidence rate.^31^ Implants placed during reconstructive surgery and in grafted bone are at high risk for ORN.^31^ However, radiation dose above 60 Grey, a one-year waiting period between irradiation and implantation, submerged versus non-submerged approaches, and implant-loading protocols did not seem to affect ORN risk.^31^ General recommendations include careful consideration of gap replacement options, prudent case selection with medical and dental optimisation, and pre-radiotherapy involvement of the restorative specialist in implant site selection.

Patients with ORN should be promptly referred to a tertiary maxillofacial unit. Management ranges from supportive care through to debridement, sequestrectomy, resection and free flaps, with or without adjuncts, such as hyperbaric oxygen (HBO) and pentoxifylline-tocopherol/pentoxifylline-tocopherol-clodronate (PENTO/PENTOCLO).^32^^,^^33^^,^^34^ Existing regional variations in use of these treatment options may be explained by differences in access and ongoing translational research.

### HBO for ORN

HBO stimulates angiogenesis, cell growth and collagen synthesis,^35^ which has been proposed to target the 'hypoxia, hypocellularity and hypovascularity' of irradiated tissues in ORN pathogenesis (Marx's 'three H' theory).^28^ Its utility in ORN treatment and prevention must be considered against its potential complications, such as barotrauma and seizures, as well as financial and time costs.^36^

Therapeutic HBO as an adjunct to surgical treatment of ORN has gained widespread adoption, but the underlying evidence remains controversial.^37^ Annane *et al.*'s 2004 trial was stopped prematurely due to worse outcomes in the HBO group.^38^ However, the 2022 DAHANCA-21/NWHHT2009-1 trials reported improved ORN healing with adjunctive HBO (70%) compared to surgical debridement only (51%).^32^ The findings were not statistically significant, which the authors attributed to the trials being under-powered; however, these promising results call for further investigations in this area.^32^

Prophylactic HBO for ORN associated with minor oral surgery is not well-supported. The 2019 HOPON trial reported a low overall ORN incidence (6%) which was similar across HBO and control groups.^39^ Previous systematic reviews have found weak to no evidence for this utility, reporting such studies to be under-powered with variable study designs.^40^^,^^41^

### PENTO/PENTOCLO for ORN

A novel combination of PENTO and/or PENTOCLO for refractory cases targets the fibroatrophic theory of ORN pathogenesis, where pentoxyfylline (phosphodiesterase inhibitor) improves tissue vascularity, tocopherol (vitamin E) scavenges free radicals and clodronate (bisphosphonate) inhibits bone resorption.^42^

Initial therapeutic use by Delanian *et al.* in 2011 revealed impressive outcomes, where all 54 refractory ORN patients in the study fully recovered over a median of nine months.^42^ However, lengthy treatment time and excessive treatment burden were drawbacks.^42^ Recent systematic reviews of subsequent observational studies have reported more modest but still considerable benefits, with a need for further robust studies.^33^^,^^34^ Furthermore, a 2020 trial by Delanian *et al.* using therapeutic PENTOCLO for post-radiotherapy brachial plexopathy showed no benefit.^43^ Ongoing trials will compare PENTOCLO with supportive care^44^ and HBO.^45^

There is emerging evidence for prophylactic PENTOCLO. Two recent retrospective reviews of 82^46^ and 110 patients^47^ who were administered PENTOCLO before extractions found an ORN incidence of less than 2%,^46^^,^^47^ compared to a baseline of 7%.^30^ Further well-structured comparisons are required to support the use of PENTOCLO in ORN prevention.

### Other adjuvant therapy for ORN

Antibiotics, being easily administered and available, are commonly used for ORN prevention despite scarce supporting evidence. Although antibiotics can reduce infection in irradiated tissues, there is no pathophysiological rationale for its use in ORN prevention. Weak evidence exists for antibiotic use, conferring a 1% absolute risk reduction in ORN compared to no antibiotics, with variable regimens reported.^30^ Antibiotics should not be used where there is no infection.

Autologous platelet concentrates which release growth factors, such as platelet derived growth factor, transforming growth factor-beta and vascular endothelial growth factor, have been proposed to improve tissue healing, with applications in bone grafts and various head and neck procedures. However, trials using platelet-rich plasma^48^ and leucocyte- and platelet-rich fibrin^49^ have shown no benefit for ORN prevention or pain scores. The use of autologous platelet concentrates for ORN is questionable.

Novel therapies for ORN, such as teriparatide (recombinant human parathyroid hormone), laser therapy and ozone have been proposed due to success in other bony pathologies, such as osteoporosis and medication-related osteonecrosis of the jaw. Teriparatide, which stimulates bone remodelling, was a successful treatment for two refractory ORN cases in 2017.^50^ Combined low-level laser therapy and antimicrobial photodynamic therapy demonstrated good mucosal healing effects in a 2018 case series.^51^ Ozone has antimicrobial and immune activation properties, and the application of topical ozone gel in a 2019 study resulted in complete mucosal healing in six of eight patients.^52^ The success of these therapies for ORN are limited to small case series, and larger, well-designed studies are necessary to establish an evidence base.

### Follow-up and discharge

Follow-up should be performed by a head and neck cancer MDT at accredited units. Patients who have successfully completed all treatment and have no active complications may be discharged back to primary dental care with an appropriate handover, regarding the patient's dental needs and indications for re-referral.^53^ General recalls every three months should reinforce preventive advice and proactively manage any oral health issues through review of the treatment history and any complications, and a comprehensive examination and thorough oral hygiene assessment.

## Conclusion

Management of patients irradiated for head and neck cancer is an excellent opportunity for preventive care. There are several proposed adjunctive therapies for the prevention and treatment of complications; however, few of these are supported by strong evidence, and regional variations in use are common. Further research is required in multiple areas, in particular pre-radiotherapy extractions, implant placement in irradiated bone, and the use of HBO and PENTO/PENTOCLO.
